# Maintaining a balance: a focus group study on living and coping with chronic whiplash-associated disorder

**DOI:** 10.1186/1471-2474-11-158

**Published:** 2010-07-13

**Authors:** Kariann Krohne, Camilla Ihlebæk

**Affiliations:** 1 Faculty of Health Sciences, Oslo University College, Oslo, Norway; 2 Research Group for Nature, Health and Quality of Life, IHA, Norwegian University of Life Sciences (UMB), Ås, Norway; 3 Research Group for Stress, Health and Rehabilitation, Uni Health, Uni Research, Bergen, Norway

## Abstract

**Background:**

There is little qualitative insight into how persons with chronic Whiplash-Associated Disorder cope on a day to day basis. This study seeks to identify the symptoms persons with Whiplash-Associated Disorder describe as dominating and explore their self-initiated coping strategies.

**Methods:**

Qualitative study using focus groups interviews. Fourteen Norwegian men and women with Whiplash-Associated Disorder (I or II) were recruited to participate in two focus groups. Data were analyzed according to a phenomenological approach, and discussed within the model of Cognitive Activation Theory of Stress (CATS).

**Results:**

Participants reported neck and head pain, sensory hypersensitivity, and cognitive dysfunction following their whiplash injury. Based on the intensity of symptoms, participants divided everyday life into good and bad periods. In good periods the symptoms were perceived as manageable. In bad periods the symptoms intensified and took control of the individual. Participants expressed a constant notion of trying to balance their three main coping strategies; rest, exercise, and social withdrawal. In good periods participants experienced coping by expecting good results from the strategies they used. In bad periods they experienced no or negative relationships between their behavioral strategies and their complaints.

**Conclusions:**

Neck and head pain, sensory hypersensitivity, and cognitive dysfunction were reported as participants' main complaints. A constant notion of balancing between their three main coping strategies; rest, exercise, and social withdrawal, was described.

## Background

Whiplash was defined in 1995 by the Quebec Task Force as a neck injury mechanism and may result in injuries within the musculoskeletal and/or neurological system. The Quebec Task Force developed a system for grading Whiplash-Associated Disorders (WAD): WAD I-II (symptoms without known pathology), III (symptoms and neurological signs), and IV (symptoms and cervical fracture and/or dislocation) [1].

Grade I and II patients represent up to 90% of "whiplash injury claims" [2]. The proportion of patients who reports pain and disability six months after the accident (i.e. chronic WAD) varies substantially between studies and countries [3,4]. However, a recent review suggests that approximately 50% of the patients with WAD will report neck pain symptoms one year after their injuries [5]. Patients with chronic WAD report high levels of neck pain, headache, and shoulder pain often accompanied by neck stiffness, dizziness, fatigue, sleeping problems, concentration problems, allergy, breathing disorders, hypertension, cardiovascular disorders, digestive disorders, depression, anxiety, and impairment in cognitive performance [6-11]. A recent study of a large population-based cohort of victims of car accidents, found that isolated neck pain was rare and that pain from multiple body areas was most commonly reported [12].

Expectations and coping styles might influence the outcome and prognosis after whiplash injuries [13]. The Cognitive Activation Theory of Stress (CATS) describes stress response as a general normal, healthy, and necessary alarm [14]. There may be a risk of illness and disease only if the arousal is sustained. The level and duration of the alarm depends on the expectancy of the outcome of stimuli, as well as the results from specific responses available for handling the situation. Therefore, the CATS model emphasizes the importance of coping as positive response outcome expectancies. This means that if the individual expects to be able to handle a situation with a positive result (coping), the activation will be short and do no harm. Kivioja et al. [15] found no evidence that early coping strategies influenced the prognosis after whiplash injuries. Others, however, found that high levels of passive coping strategies are associated with a slower recovery after whiplash injury [16,17], and that certain coping strategies for pain, such as catastrophizing, is associated with increased risk of disability, and that the importance of coping strategies seem to increase over time [18-21]. In general, there is considerable controversy as to the importance of psychological factors for developing chronic WAD [22].

The importance of insight into coping strategies has been emphasized for chronic pain patients such as fibromyalgia [23], tension-type headache [24], chronic back pain [25], and chronic temporomandibular disorder [26]. However, there is little qualitative insight into the ways persons with chronic WAD cope on a day to day basis. Such insight may provide the clinician with a better understanding of lay health recourses, and, possibly, provide a better starting point for suggesting strategies or discussing potentially maladaptive strategies to patients suffering pain following whiplash. Furthermore, Russell & Nicol [27] suggested that WAD patient recovery may be increased if the clinicians better understand patient experiences. In the present study we identify what is described as dominant whiplash symptoms, and the behavioral strategies used to cope with WAD.

## Methods

The focus group method was chosen for this study. Focus groups are considered particularly suitable to render in-depth information about a concept or an issue, and learn about people's experiences [28]. The benefit of the method is that it creates room for group dynamics enabling participants to express themselves in a flexible discussion [29] using their own words and according to own priorities. Several studies have used the focus group method to highlighting strategies for coping with various chronic pain disorders [23,24,30]. The analytic framework adopted in this study is phenomenology giving priority to the participants' descriptions of their lived experiences.

### Recruitment and Participants

The study was approved by the Regional Committee for Medical Research Ethics in Norway. Participants were recruited by distributing a recruitment email to members of a local branch of the Norwegian Whiplash Association and another smaller whiplash patients' activist organization. Individuals volunteering to participate contacted the researcher by email or telephone, and arrangements concerning the scheduling of the focus group sessions were made. The researchers initially aimed at including persons defining themselves as WAD grade I or II, but the individuals volunteering to participate revealed that WAD was not a familiar concept or grading system to them. It was, therefore, decided, in keeping with the criteria of WAD I-II, to include individuals who stated to have symptoms, but without known pathology. One individual decided not to participate after being briefed on this, since he had undergone neck surgery. Fourteen participants (six men and eight women) agreed to participate and were divided into two focus groups (group sizes eight and six).

### Group characteristics

Participants' age ranged from 39 to 63 (average = 51). All were of Caucasian ethnicity, living in the south-eastern parts of Norway. Eight reported highest education to be university or college. Participants reported that their accidents happened from 1981 to 2006. Eleven participants claimed trauma to the neck following a car accident. Three participants were injured following accidents related to sports or falling. Three participants had experienced several accidents causing multiple traumas to the neck. Common in their accounts was the experience of neck pain and stiffness directly after the accident. All returned to work after the accident, but they explained that, over time, a constant worsening of symptoms made them give up or reduce their employment. At the time of the interviews one participant worked full time, two worked part time, and 11 did not work at all. In search of symptom relief all reported to have tried numerous medical treatment schedules, private and governmental rehabilitation institutions, and training schemes. Difficulties maintaining a stable family life, and lawsuits following disability claims were also mentioned.

### Focus Group Interviews

The 90-minute sessions took place in a University meeting room in March and April 2007. At the beginning of each focus group participants signed a consent form, and provided written information on age, education, current work situation and the medical and therapeutic treatment schedules undertaken due to their condition. The moderator (CI) commenced the interview sessions by informing about the study's purpose, limits of confidentiality, and their right to withdraw according to the Helsinki Declaration. The interview was guided by eight open-ended questions on whiplash-associated symptoms, life situation, and medical treatment. The interview guide was generated following comprehensive review of the current literature and designed to enhance group interaction and group conversation. The moderator encouraged participants to discuss questions freely, and in both sessions the level of participation was high. The groups self-facilitated so that all participants were included and active in the discussions [31]. For the purpose of this paper's focus on symptoms and coping strategies, three questions were relevant:

What is it like to live with a whiplash injury?

How does your whiplash injury affect your daily life?

What do you do, in your everyday life, to handle the effects of your whiplash injury?

First question was an introductory question designed to engage participants in the topic, the latter two were key questions. The sessions were observed and audio-taped by the first author (KK), who also posed complementary questions towards the end of each session. Participants received a 500 Norwegian kroner honorarium. Both sessions were transcribed verbatim by secretarial staff.

### Analysis

Systematic Text Condensation [32], a modified version of Giorgi's phenomenological approach [33], was applied in the analysis of the data. Four steps were followed: (i) Transcripts were read to gain a contextualized impression of the discussions, and preliminary themes chosen. (ii) Units of meaning were identified and coded. (iii) The meaning in the coded groups was condensed. (iv) Descriptions were then summarized to establish concepts reflecting the dominating symptoms and the coping strategies used by the participants. The analysis showed a high level of theme saturation between the groups. The qualitative findings are presented as descriptive summaries under the two subheadings *Dominant symptoms *and *Behavioral strategies*, and illustrated by quotes from the transcripts. Quotes are translated from Norwegian by the first author, and coded with group and participant number. The findings are in the discussion interpreted within the CATS model [14].

## Results

### Dominating symptoms

Participants reported three major symptoms affecting their daily life: Cephalic and cervical pain, sensory hypersensitivity, and cognitive dysfunction. These three were repeatedly portrayed within a hierarchal framework: Cephalic and cervical pain was communicated as predominant, and words like *intolerable *and *indescribable *were used to describe everyday life in the periods of constant pain. Periodically, the pain experience was constant both day and night, and consequently sleeplessness was common. Several participants revealed that they used painkillers and anti-inflammatory medication to reduce the pain, but also to be able to sleep. One participant described the omnipresence of the cephalic and cervical pain in the following way:

*2/3. The first thing is the headache. It's the main thing the whole way. You'll always feel that pressure. Can't relax - you're never able to lower your shoulders. There's always that little murmuring in the head*.

Another participant elaborates, and adds the aspect of the varying intensities of the pain:

*1/7. [F]or me the worst and the most destructive part is the pain ... that constant headache. It kind of manifests itself in the neck and spreads up throughout the head - and it seems to have different intensities all depending on how close to the eyes it gets*.

In addition to the cephalic and cervical pain, and the resulting limited motor functions in the neck and shoulder area, the participants also stressed that sensory dysfunctions affected their lives in a major way. Hypersensitivity to light and sound was a large everyday challenge. Music, noise from children, or heavy machinery operating nearby were experienced as intolerable;

*2/2. I had *[teenagers] *who played music, and they weren't allowed to exist because they had their mum to consider. It's been unendurable not to handle sound, not to handle light while dealing with all the other stuff...*

The participants also claimed to have reduced eyesight and hearing following their WAD. They experienced tunnel vision and believed firmly that their judgment of distance had weakened. The hearing problems were not only experienced by participants, people close to them also made remarks:

*2/1. [M]y husband says,"[G]et your hearing checked out"- but I've had it done and it's 100%. Clearly, it's got to do with me concentrating so much, and spending so much energy concentrating that I actually close out other things*.

This quote not only underlines how the experience of reduced hearing is confirmed by others; it also reveals the participant's own causal interpretation. She considers her reduced hearing a consequence of *spending so much energy concentrating *on some things that she closes out everything else. Her interpretation is confirmed by the other participants as the essence of the cognitive dysfunction-related complaints was described as reduced ability to concentrate and reduced memory. One participant describes the experience and the consequences of living with reduced ability to concentrate:

*1/7. Reduced ability to concentrate - no doubt! You just feel so isolated. You can't participate in festivities. (...). You're not able to sit in a crowd, attend meetings ... and differentiate who's actually talking. Well, it can be done, [but] if they start to talk simultaneously - like people do in social gatherings - then it's completely impossible! You just have to get out of there, and you'll get sick, very sick with dizziness and a nasty wooziness in the head - it's difficult to explain*.

The cognitive dysfunction portrayed here is perceived both as a pain trigger in itself and as a barrier to participate in social life. Further discussions among the participants on this topic revealed that cognitive dysfunctions were also considered a major barrier to employment. Reduced memory, as well as reduced capacity to read, write, or work in front of a computer made it difficult to keep professional skills updated.

1/3. I've been holding on to my job for dear life; on sick-leave and working, on sick-leave and working (...) eventually I had to give it up. I realize that I'll never return to work (...). Since I'm not able to read or learn anything new now - I just don't know what to do...

Based on the intensity of the symptoms, participants divided everyday life into good periods and bad periods. In good periods, the symptoms were perceived as manageable, but in bad periods the symptoms intensified and took control over the individual. The pain in bad periods could lead to frustration, anger, and depression. A participant explains:

*2/5. I'm on anti-depressives these days because I got sick again - from all this pain. I'm pretty low psychologically speaking. I sort of hit rock bottom right before Christmas (...). And I'm struggling to get back up again*.

### Behavioral strategies

Participants in both groups felt that as whiplash patients they were ignored by the medical expertise. Left to themselves they started to develop coping strategies to cope with their symptoms. One participant described this process:

*2/6. So when you discover that there are no miracle cures for the neck ... well, then you just have to deal with it and develop your own strategies. (...). Because you won't get any information - you'll have to figure it all out for yourself*.

Finding suitable strategies were described as a process of testing the available medical and alternative treatments, trying out different training schemes, and self-experimenting. Subsequently, they were able to develop dynamic coping strategies, i.e. coping strategies constantly adjusting to the fluctuating intensity of their symptoms and the shifts between good and bad periods. The process of discovering effective coping strategies entailed significant life style changes:

*1/8. You need to (...) learn how to adjust your life in relation to stress, level of ambition, barriers, and all that. Luckily you get better at it, but you do have to settle for a different level than you originally planned*.

Another participant pinpointed some of the specific consequences these life style changes had on his life:

*1/7. Everyday life is very controlled. It's training and resting, and avoiding everything that might trigger a worsening. Well, I have given up all hobbies - boating and hunting ... forget it. (...). So it's necessary to be very controlled, but we should get used to it because it's worth it. The weeks that you manage to be slavishly and work only when *[the pain] *allows you to, that gives a great sense of satisfaction to manage that, right? (...) Even though I miss it all*.

Maintaining good periods, and avoiding or shorten the bad periods, were portrayed as the main goals for their coping strategies. The participants used the term "balance" to describe the intention behind their self-initiated strategies; *you have to maintain a balance all the time - and not exaggerate anything (2/5)*. They claimed that maintaining a balanced life was imperative to be able to function with their symptoms.

The most important factor in maintaining a balanced life was the possibility to rest in calm surroundings. This meant that they would decline invitations to social gatherings, or that they would leave the house if it was too noisy. Rest was used to prepare for events:

*2/3. If I'm planning anything in the near future then it's three days indoors first ... to charge up my batteries. No social life, no light, or sounds out of the ordinary, no computer, no TV, or anything like that for three days - then I can participate*.

Rest was also used to unwind and regain control over the pain that occurred after being exposed to social activities, reading, music, driving, or flying. *If you're not able to recuperate, then you'll get it back twice as hard! You're just making your own hell! (1/6)*

Another strategy was exercise. For the majority of participants exercise was a dominating part of everyday life:

*2/1. I use a large proportion of the day - before noon - to exercise. When I've exercised I need to relax - if not I'll be in a bad state in the afternoon*.

The ones who found training methods with good effect used these as "self-medication", since they were able to reduce their original use of painkillers when the exercise had effect. *I'll rather take a hike in the woods. That's my medication (1/2)*. The exercise sessions described by the participants were training therapy using elastics, walking, hiking, swimming, or other forms of self-training. Participants maintained that only a couple of days without training would have a negative impact on pain intensity. Regularity of exercise intensity and tempo was consequently important for the desired effect. A few participants reported that even simple training exercises would trigger pain. *I have tried lots of training schemes (...). But what I experience is that it triggers headache. (4/2)*. Consequently physical activity was avoided and these participants preferred to use rest to maintain a balance.

Rest and exercise were described as both time and mind consuming strategies, and, throughout the focus group sessions, it was understood that they required various levels of social withdrawal from the individual pain sufferer. However, participants stated that social withdrawal was an important coping strategy in itself. Keeping to oneself was an efficient way to avoid potential pain triggers and maintain good periods:

*1/8. I discovered that I became more and more antisocial. When I came home from work or ... I just preferred to be by myself, (...) that was my way of coping. It was a scary discovery*.

## Discussion

### Summary of main findings

The aim of the present study was to identify dominant whiplash symptoms, and the behavioral strategies used to cope with these. Participants stated dominating symptoms to be neck and head pain, sensory hypersensitivity, and cognitive dysfunction. In describing their dominating symptoms participants gave emphasis to a fluctuating level of pain - dividing their life into what they described as a repeating cycle of good and bad periods. To cope with these symptoms, maintaining the good periods and avoiding or shorten the bad periods, they used rest, exercise, and social withdrawal. Participants expressed a constant notion of alternating or *balancing *between these coping strategies following the intensity of symptoms, or the expectancy of participating in situations or events that might trigger pain.

### Strengths and limitations of the study

Proactively, participants responded to the recruitment email sent to members of two small organizations. Recruiting from small organizations may affect the transferability of our findings since the participants may not represent the typical whiplash patient. On the other hand, it may be that these individuals provided the study with more in-depth information since membership in a whiplash organization implies a proactive and a well thought out stance on own life situation. The information provided by the participants on symptoms is seen in other studies on whiplash patients [34-36]. The concept of balancing everyday life using coping strategies finds a parallel in a study by Slettbakk *et al. *on tension-type headache sufferers [24]. Still, one should beware that accounts presented reflect participants' experiences and actions, and that qualitative findings are not meant to be applicable to a general population [37].

In this study lay health resources are accounted for, and since we neither intended to evaluate the level of pain following symptoms nor the effect of the strategies, we did not assign a biomedical value to participants' condition or their strategies [24]. The essence of the study was the phenomena discussed. Whiplash symptoms and the issue of chronicity were, hence, not medically documented, but they were discussed and recognized in the groups.

### Whiplash symptoms: fluctuating pain

Participants reported severe neck and head pain, sensory hypersensitivity, and cognitive dysfunction as their main complaints. These symptoms are reported in several other studies [3,9,34-36]. The pain was not described as being on a permanent level, but, rather, as fluctuating from a severe and intolerable level of pain to a more manageable pain. This fluctuating pattern was by the participants described as having bad and good periods. The symptoms were closely connected together as one could cause the onset of the other. Such a pattern of fluctuating pain and incapacity which is difficult to predict and manage, has also been reported in other studies on chronic pain [23,38,39], and it affects not only own health, but also family life and social activities [39,40].

### Behavioral strategies to cope with symptoms in everyday life

A main finding in this study was how participants divided everyday life into good and bad periods, and how they adjusted their coping strategies according to this. Participants expressed a constant notion of alternating between or balancing their three main coping strategies; rest, exercise, and social withdrawal. If the balance - *viz *choosing and implementing the best strategy - was not maintained pain could be triggered or bad periods prolonged. The strategies were, primarily, chosen based on the intensity of symptoms, but it was also reported in the focus groups that the same strategies, mostly rest and social withdrawal, were used as means to prepare for, or unwind from, possible pain triggering situations or events.

Lazarus & Folkman's [41] cognitive-phenomenological model of stress and coping discriminates between active and passive coping strategies. Active or problem-focused strategies are used to target the source of stress and reduce it, whereas passive or emotional-focused strategies are mostly concerned towards adapting to the stress or problem. Most of our participants used exercise, i.e. active coping strategies in good periods as they experienced that it reduced pain. Passive coping strategies, such as rest and social withdrawal, were mostly used to endure pain and to maintain the important balance as the participants were afraid of provoking bad periods. Social withdrawal may be interpreted as a direct consequence of their lifestyle changes, but participants also perceived it as a coping strategy *per se *- primarily used to avoid triggering the pain brought on by being exposed to noise, concentrating, or focusing too much.

Contrary to the Lazarus use of coping strategies, the Cognitive Activation Theory of Stress (CATS) suggests that it is not the strategy or way of coping that is the most important issue, but the expectancy of the result [14]. In the good periods our participants engaged in behaviors they expected to improve their circumstances - regular exercise being the most important one. The use of rest and social withdrawal were also used in good periods as a way of 'charging the batteries' for special events. The participants expected and experienced positive results of these behaviors, i.e. coping in the terminology of the CATS model [14].

However, what participants referred to as bad periods was characterized by unremitting pain often leading to frustration, depression, and social isolation. The participants had to socially withdraw and rest during these periods. In bad periods they felt that the symptoms took control of them, and that there was nothing they could do but rest and wait for a good period. Several participants experienced depression due to their situation. Within the CATS model helplessness or hopelessness develops when there is either no relationship or a negative relationship between what the individual attempts to do and the outcome. This may lead to sustained arousal, which, in turn, could lead to illness and disease such as depression [14] and chronic fatigue syndrome [42].

The participants expressed that, to some degree, they could control or predict bad periods; consequently they tried to balance their life to avoid these periods. The constant notion of trying to balance; the restrictions and sacrifices behind their coping strategies took its toll on everyday life. The pattern of coping strategies described in this study was in accordance with other studies on patients with chronic pain [24,40,43], and was perceived as effective for our participants. However, it could be discussed whether or not the behavioral strategies, even though they might lead to positive response outcome expectancies, are adaptive or not. Most participants expressed a wish to be able to participate in working life. Nevertheless, only three in 14 participants had been able to maintain a work situation. So, although the strategies used were considered the most beneficial - or the only way to adjust their life, it is questionable whether or not they led to progress or just maintained the pattern of alternating good and bad periods. Knowledge of patients' self-initiated coping strategies may give the clinician a better understanding of the patients' frame of reference; how they organize everyday life to cope with their problems, and, accordingly, establish a better starting point for discussing potentially maladaptive strategies.

## Conclusions

Participants reported severe neck and head pain, sensory hypersensitivity, and cognitive dysfunction as their main complaints. To cope with these complaints, and their fluctuating nature, three main strategies were used; rest, exercise, and social withdrawal. The participants portrayed that maintaining a balance between these coping strategies helped control the pain.

## Competing interests

The authors declare that they have no competing interests.

## Authors' contributions

CI conceived of the study. KK developed its methodology. KK and CI took part in the collection of data. KK analyzed the data. Both authors drafted the manuscript, reviewed and edited drafts of the manuscript. All authors read and approved the final manuscript.

## Pre-publication history

The pre-publication history for this paper can be accessed here:

http://www.biomedcentral.com/1471-2474/11/158/prepub
